# A thank you note to our peer reviewers (2023)

**DOI:** 10.1002/jia2.26335

**Published:** 2024-07-24

**Authors:** Kenneth H. Mayer, Annette H. Sohn, Marlène Bras

**Affiliations:** ^1^ The Fenway Institute and Harvard Medical School Boston Massachusetts USA; ^2^ TREAT Asia amfAR ‐ Foundation for AIDS Research Bangkok Thailand; ^3^ The International AIDS Society Geneva Switzerland

The *Journal of the International AIDS Society* (JIAS) would like to express our gratitude to the peer reviewers who contributed to reviewing articles for the journal in 2023. Their time and expertise are crucial to upholding the quality of this publication, and we are thankful for their engagement.

We also wish to extend our appreciation to the JIAS Editorial Board members, Deputy Editors, statistical experts and Ethical Committee members for their valuable contributions in assessing and reviewing articles submitted to the journal.

Kenneth Mayer, co‐Editors‐in‐Chief

Annette Sohn, co‐Editors‐in‐Chief

Marlène Bras, Executive Editor

Aaloke Mody

Adam Trickey

Adekemi Sekoni

Aditi Ramakrishnan

Aditya Subhash Khanna

Alana T. Brennan

Albert Liu

Alex Dubov

Alex Keuroghlian

Alex Viguerie

Alexander Adia

Alexandra C. Vrazo

Allan Maleche

Allanise Cloete

Allison McFall

Amelia M. Stanton

Amy Zheng

Anatole Menon‐Johansson

Andrea Jane Low

Andrew Hill

Andrew McAuley

Andrew Prendergast

Angela Bengtson

Aniruddha Hazra

Ann Gottert

Anna Bershteyn

Anna Grimsrud

Anthony Fojo

Antons Mozalevskis

Anupam Garrib

Aoife Doyle

April D. Kimmel

Ariane van der Straten

Ashley Lacombe‐Duncan

Augustine Talumba Choko

Bankole Olatosi

Benjamin Brown

Benjamin H. Chi

Bernadette Kina Kombo

Bernard Surial

Bill G. Kapogiannis

Bindiya Meggi

Brandon Guthrie

Brenda Hoagland

Brennan Cebula

Brian Zanoni

Bronwyn Elizabeth Bosch

Brooke E. Nichols

Bruce Richman

Caitlin Dugdale

Camille Cioffi

Carla Pires

Carmen Logie

Carol S. Camlin

Carol Strong

Caroline De Schacht

Caroline Foster

Carolyn Bolton‐Moore

Carolyn Lauckner

Catherine Godfrey

Catherine Lesko

Cheryl Case Johnson

Chris Collins

Christian Kraef

Christina Psaros

Chutima Suraratdecha

Claudia Estcourt

Clemens Benedikt

Collins Iwuji

Daisuke Mizushima

Daniel Fierer

Danielle Resar

Darrell Tan

David Allen Roberts

David B. Hanna

David Dunn

David Hoos

David V. Glidden

Dean Murphy

Deanna Kerrigan

Debrah Boeras

Denis Nash

Denise Jacobson

Didier Ekouevi

Dobromir Dimitrov

Donn Colby

Doris Chibo

Dorlim Antonio Moiana Uetela

Dvora Joseph Davey

Edinah Mudimu

Elaine J. Abrams

Elenore P. Bhatraju

Elijah Kakande

Elizabeth T. Knippler

Elliot Raizes

Elona Toska

Emily Chasco

Emily Hyle

Emma Kalk

Erica N. Browne

Erin Graves

Erin Wilson

Estevão P. Nunes

Esther C. Atukunda

Fiona Burns

Florence Anabwani

Francoise Renaud

Fumiyo Nakagawa

Gabriel Chamie

Gbolahan Ajibola

Gene Morse

George Ayala

George Siberry

Gesine Meyer Rath

Giang Thi Hoang

Gillian Dougherty

Giuliana Jacqueline Morales

Guan‐Jhou Chen

Habib Ramadhani

Halima Dawood

Hanne Zimmermann

Heather Bailey

Heather Pines

Heather‐Marie Schmidt

Helena Rabie

Homaira Hanif

Hong Chen

Ian Hodgson

J. Joseph Lawrence

Jack DeHovitz

Jack Stone

Jacklyn D. Foley

James Ayieko

James Carlucci

Jane Tomnay

Jason W. Mitchell

Jasper S. Lee

Javier Rodriguez‐Centeno

Jean de Dieu Tapsoba

Jean‐Pierre Routy

Jennifer Cocohoba

Jennifer M. Belus

Jennifer Sherwood

Jeremy Penner

Jerome Timothy Galea

Jessica E. Haberer

Jessica J. Justman

Jienchi Dorward

Jing Zhang

Joel Msafiri Francis

Joep J. Van Oosterhout

John Chiosi

John M. Humphrey

John Stover

Jonathan Ross

Jose A. Bauermeister

Joseph Kagaayi

Julia Raifman

Julia Rohr

Julie Pulerwitz

Junko Tanuma

K. Rivet Amico

Kai J. Jonas

Kaitlyn Atkins

Karin Hatzold

Karl Technau

Karsten Lunze

Kassem Bourgi

Kate Wilson

Katerina Christopoulos

Katherine Horton

Kathleen MacQueen

Kathrine Meyers

Katrina Frances Ortblad

Kawango Agot

Kelika Konda

Kelli O'Laughlin

Kerry Mangold

Kim A. G. J. Romijnders

Kim Steegen

Kimberly Hook

Kimberly Powers

Kostyantyn Dumchev

Kristen A. Stafford

Landon Myer

Lara Vojnov

Larry W. Chang

Laura Beres

Laura C. Nyblade

Laura Oyiengo

Laura Waters

Lawrence Long

Lee Fairlie

Linda J. Koenig

Lisa Lynn Abuogi

Lisa O'Brien

Lise Jamieson

Lorena de la Mora

Lori Miller

Louise Kuhn

Luann Hatane

Luis Sagaon‐Teyssier

Lynda Myra Oluoch

Lynne Wilkinson

Maarten Reitsema

Madeleine Goldstein

Marguerite Thorp

Mariano Kanamori

Marilyn Lake

Mario Enrico Canonico

Marjorie Opuni

Mark Sonderup

Mary Ann Davies

Mary Jane Rotheram‐Borus

Mary Jo Trepka

Mary Tumushime

Mathieu Maheu‐Giroux

Matthew Hogben

Matthew D. Hickey

Maxime Inghels

Megan Rose Curtis

Mehri McKellar

Mélanie Plazy

Melissa Mugambi

Melissa Schnure

Michael Traeger

Michele Montandon

Michele P. Andrasik

Michelle Ann Bulterys

Milosz Parszewski

Minh Le

Mmamapudi Kubanje

Molly Rosenberg

Monisha Sharma

Moses H. Bateganya

Mostafa Dianatinasab

Musonda Simwinga

Nancy Puttkammer

Natalia Lorna Laufer

Natella Rakhmanina

Nathan P. Ford

Navindra E. Persaud

Nelson Kalema

Ngai‐sze Wong

Nicolette M. du Plessis

Nikki Cockern

Nikos Pantazis

Nivedita Bhushan

Nobubelo Ngandu

Nomathemba Chwoneso Chandiwana

Norma Ware

Nyikadzino Mahachi

Obinna Ikechukwu Ekwunife

Ola Farid Jahanpour

Olga Morozova

Oliver T. Stirrup

Pablo Noel Perez Guzman

Pamela Bachanas

Parastu Kasaie

Patience Nyakato

Peter F. Rebeiro

Peter Havens

Phillip Keen

Pierre DeBeaudrap

Poyao Huang

Praphan Phanuphak

Priscilla Ruvimbo Tsondai

Rachel Gruver

Radhika Sundararajan

Rebecca Zimba

Rena Patel

Reshmie Ramautarsing

Richard Harding

Robert Remien

Robin MacGowan

Roger Detels

Roger Ying

Romain Palich

Rose Pollard Kaptchuk

Rupa Patel

Sajida Julius Kimambo

Samantha Yeager

Samuel A. Jenness

Sarah B. Puryear

Sarah Bernays

Sarah K. Calabrese

Sarah‐Jane Anderson

Scholastic Ashaba

Scott Dryden‐Peterson

Serena Rajabuin

Seth Frndak

Sharmistha Mishra

Sharon Louise Hillier

Sheetal Kassim

Shelley N. Facente

Shenaaz Pahad

Sheree Renae Schwartz

Sherrie Kelly

Shobna Sawry

Sinead Delany‐Moretlwe

Siobhan Crowley

Sita Lujintanon

Siyang Xia

Solange Baptiste

Sophie J. S. Pascoe

Souleymane Diabate

Steffanie Strathdee

Stella Zawedde Muyanja

Stephan Rabie

Stephen Okoboi

Steven Meanley

Steven Safren

Susan Blair Timberlake

Susan Buchbinder

Susan Marie Graham

Susanne Dam Nielsen

Sylivia Nalubega

Sylvie Deuffic‐Burban

Sylvie Naar

Takashi Muramatsu

Tali Cassidy

Tampose Mothopeng

Tamsin Kate Phillips

Tanuja N. Gengiah

Taraz Samandari

Tessa Goetghebuer

Thanyawee Puthanakit

Thiago S. Torres

Thibaut Davy‐Méndez

Thomas Sumner

Thorkild Tylleskär

Tiffany R. Phillips

Todd M. Pollack

Tom Ellman

Tonderai Mabuto

Tonia Poteat

Tracey Naledi

Tristan J. Barber

Unmesha Roy Paladhi

Valentina Cambiano

Van Nghiem

Venkatraman Chandra‐Mouli

Vincent J. Wong

Vincent Joseph Tukei

Virginia A. Fonner

Virginia Macdonald

Virginia McKay

Vita W. Jongen

Vundli Ramokolo

Vuyolwethu Magasana

Weiming Tang

Whitney Irie

Yijia Li

Yogan Pillay

Yong Gun Lee

Zabrina Brumme

Zixin Wang

